# Surface-Based fMRI-Driven Diffusion Tractography in the Presence of Significant Brain Pathology: A Study Linking Structure and Function in Cerebral Palsy

**DOI:** 10.1371/journal.pone.0159540

**Published:** 2016-08-03

**Authors:** Lee B. Reid, Ross Cunnington, Roslyn N. Boyd, Stephen E. Rose

**Affiliations:** 1 The Australian e-Health Research Centre, CSIRO, Brisbane, Australia; 2 School of Psychology and Queensland Brain Institute, The University of Queensland, St Lucia, Brisbane, Australia; 3 Level 6, Queensland Cerebral Palsy and Rehabilitation Research Centre, Children’s Health Research Centre, School of Medicine, The University of Queensland, Brisbane, Australia; University of North Carolina, UNITED STATES

## Abstract

Diffusion MRI (dMRI) tractography analyses are difficult to perform in the presence of brain pathology. Automated methods that rely on cortical parcellation for structural connectivity studies often fail, while manually defining regions is extremely time consuming and can introduce human error. Both methods also make assumptions about structure-function relationships that may not hold after cortical reorganisation. Seeding tractography with functional-MRI (fMRI) activation is an emerging method that reduces these confounds, but inherent smoothing of fMRI signal may result in the inclusion of irrelevant pathways. This paper describes a novel fMRI-seeded dMRI-analysis pipeline based on surface-meshes that reduces these issues and utilises machine-learning to generate task specific white matter pathways, minimising the requirement for manually-drawn ROIs. We directly compared this new strategy to a standard voxelwise fMRI-dMRI approach, by investigating correlations between clinical scores and dMRI metrics of thalamocortical and corticomotor tracts in 31 children with unilateral cerebral palsy. The surface-based approach successfully processed more participants (87%) than the voxel-based approach (65%), and provided significantly more-coherent tractography. Significant correlations between dMRI metrics and five clinical scores of function were found for the more superior regions of these tracts. These significant correlations were stronger and more frequently found with the surface-based method (15/20 investigated were significant; R^2^ = 0.43–0.73) than the voxelwise analysis (2 sig. correlations; 0.38 & 0.49). More restricted fMRI signal, better-constrained tractography, and the novel track-classification method all appeared to contribute toward these differences.

## Introduction

Cerebral palsy is a term describing a group of permanent disorders of movement and posture owing neurological insult, or non-progressive disturbances of neurological development, that are presumed to have occurred by the time of birth [1]. The majority of cases are due to periventricular white-matter damage that is assumed to occur during the third trimester [2–4]. Children with cerebral palsy often have learning impairments, and are restricted in both their mobility and ability to participate in everyday activities [5]. As with disorders caused by acquired brain injuries, current rehabilitative efforts are hampered by a limited understanding about the relationship between brain structure, function, and brain pathology [6]. In cerebral palsy, this picture is complicated further by ongoing neural development that occurs during childhood. Structural MRI is one modality that has been utilised to investigate cerebral palsy, but relationships between gross structure and functional outcomes are not straightforward, and have proven difficult to ascertain [7].

Diffusion MRI (dMRI) is an increasingly popular modality that is used to investigate links between white matter (WM) injury and clinical outcomes. A number of dMRI studies investigating WM injury in cerebral palsy have reported relationships between microstructural characteristics of the cerebrospinal tract and functional outcomes [6,8]. In unilateral cerebral palsy, questions still remain as to whether the primary contributor to upper-limb motor impairment is damage to corticomotor tracts or associated damage to the thalamocortical sensory pathways [9,10]. Although standard analyses might have the potential to provide answers to these questions, such approaches are often difficult or impossible to perform on moderately impaired children with cerebral palsy due to the presence of pathology and potentially-atypical brain organisations.

**Fig 1:**
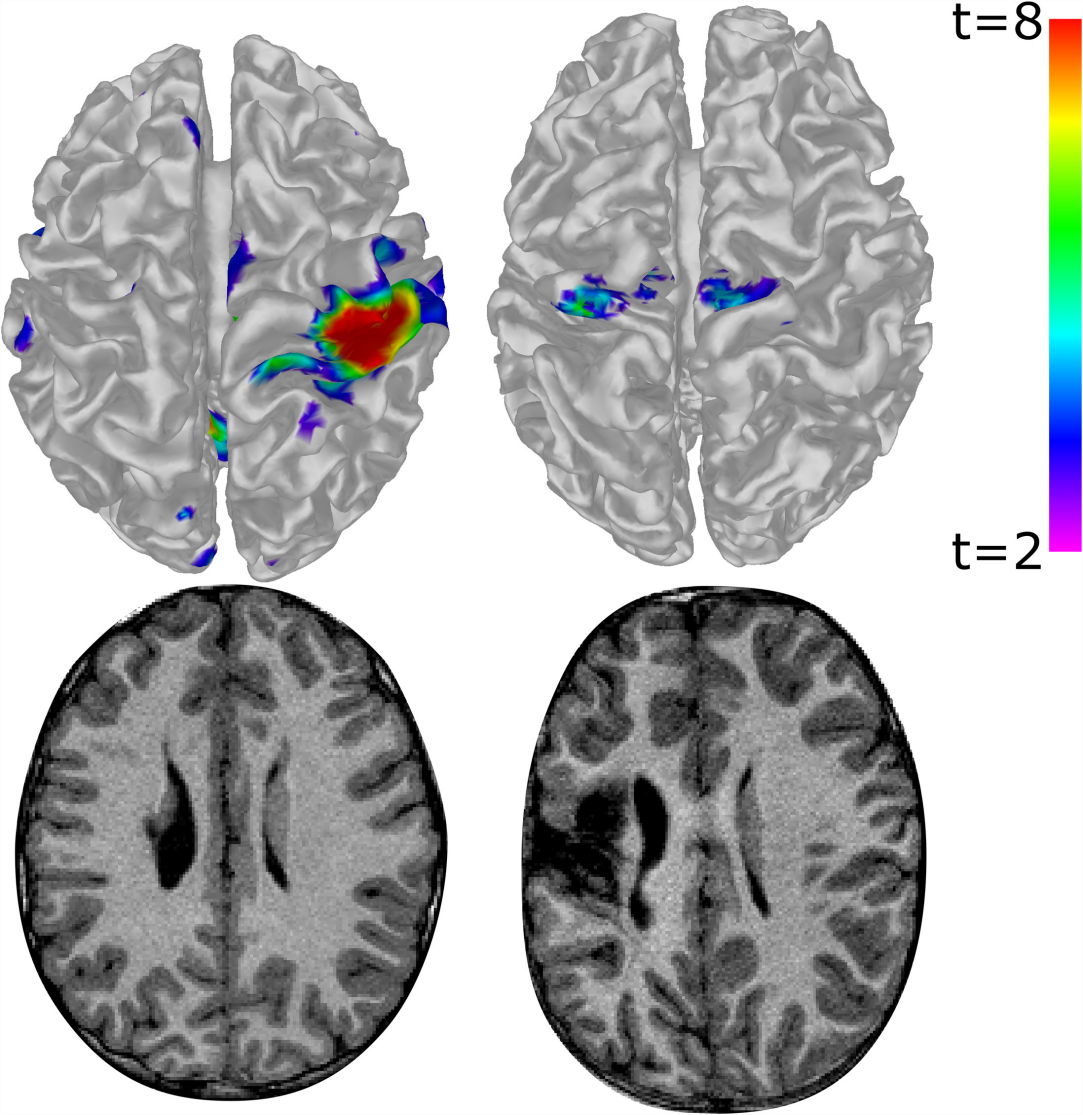
Functional MRI activation for a hand tapping task (top), and corresponding T1 images (bottom) for two children with unilateral cerebral palsy. The top-left of each image represents the anterior-left of each brain. Left Column: a higher-functioning child tapping their less-impaired (right) hand. Robust unilateral activation can be seen in the 'typical' hand knob location of the sensorimotor cortex, as may be expected from a typically developing child. Right Column: a child with higher degrees of brain damage tapping their more-impaired (left) hand. Activation appears more medially, bilaterally, and anteriorly; predominantly in the assumed supplementary motor areas rather than in the typical hand-knob locus. These differences may illustrate potentially abnormal brain organisation occuring in response to the early-life brain injury sustained. Images are not to scale.

Most diffusion analyses provide metrics that can be used to make inferences about the brain’s underlying microstructure. When studying motor function, such approaches require robust delineation of cortical motor regions and/or subregions (such as the hand knob). Cortical parcellation performs this role reliably in healthy individuals, but problems often emerge when applying analyses to individuals with brain pathology. These problems are twofold. Firstly, tools such as Freesurfer (http://surfer.nmr.mgh.harvard.edu) often fail to delineate the cortical surface when pathology or abnormal morphology is present. For example, in our experience, Freesurfer fails to delineate this surface acceptably in around 30% of children and adolescents with mild-to-moderate cerebral palsy, even after repeated manual adjustment. Consequently, the majority of studies measuring dMRI metrics in cerebral palsy (and similar conditions) so far have relied on manual-parcellation [8]. Unfortunately, manual parcellation is prone to bias or human error and is extremely time consuming, especially when pathology is present. The second issue is that cortical parcellation, both automated and manual, may be invalidated by functional reorganization that occurs in response to pathology (Fig 1). Using coarser regions of interest (ROIs; such as the entire sensorimotor cortex) might improve confidence that tissue that is functionally-relevant to the hypothesis is included, but will likely also include tissue that is functionally irrelevant to the hypothesis at hand. Tracking towards the surface using multiple hand-drawn ‘include’ ROIs is an alternative approach. This can often identify the corticomotor tracts reasonably, but is unable to reliably locate specific subregions of interest (such as those innervating the hand knob) and in a clinical situation often results in tracks that fail to reach the cerebral surface.

In situations where specific regions of interest are known a-priori, the need for cortical parcellation can be circumvented through the use of task-based functional MRI (fMRI) [6,11]. In such a method, areas of significant fMRI activation are used as seeding regions for diffusion tractography. This method has been used in several studies of healthy subjects [11], by utilising standard voxel-based software packages such as SPM (http://www.fil.ion.ucl.ac.uk/spm/software) and MRTrix [12]. Standard fMRI analyses include an appreciable amount of implicit and explicit smoothing, which can artificially increase the size of the detected activation beyond its true boundaries [6,13]. Additional smoothing also takes place when fMRI images are converted into dMRI seed images (i.e. reslicing and/or expanding activation ROI into WM). This is not usually a meaningful issue in standard fMRI interpretation, but when expanded ROIs are used for tractography seeding or filtering, this issue might result in the inclusion of irrelevant WM tracts. Applying binary inclusion/exclusion ROIs to tractography may filter out some of these tracks, but this method relies totally on assumptions of brain connectivity patterns that may not hold true in participants with pathology. The challenge of creating fair ROIs for such filtering that do not filter or miscategorise legitimate plasticity can be as difficult as manual parcellation, and is prone to the same challenges we have already discussed.

One method that can improve the specificity of fMRI-determined activation is surface-based smoothing [14]. That is, explicitly smoothing the data along the brain’s topology, rather than in image space. Theoretically, this method could constrain fMRI activation legitimately, providing a tighter seed for tractography that circumvents having to rely on highly-accurate individually-tailored ROIs for track filtering. Standard pipelines that use this method (e.g. FS-FAST), however, will also still include some implicit cross-sulci smoothing during motion correction, projection between surface and voxel-based representations, and subsequent conversion into dMRI-seed ROIs. More importantly, these methods rely on Freesurfer-based meshes that are often impossible to generate when moderate pathology or abnormal morphology is apparent.

**Fig 2:**
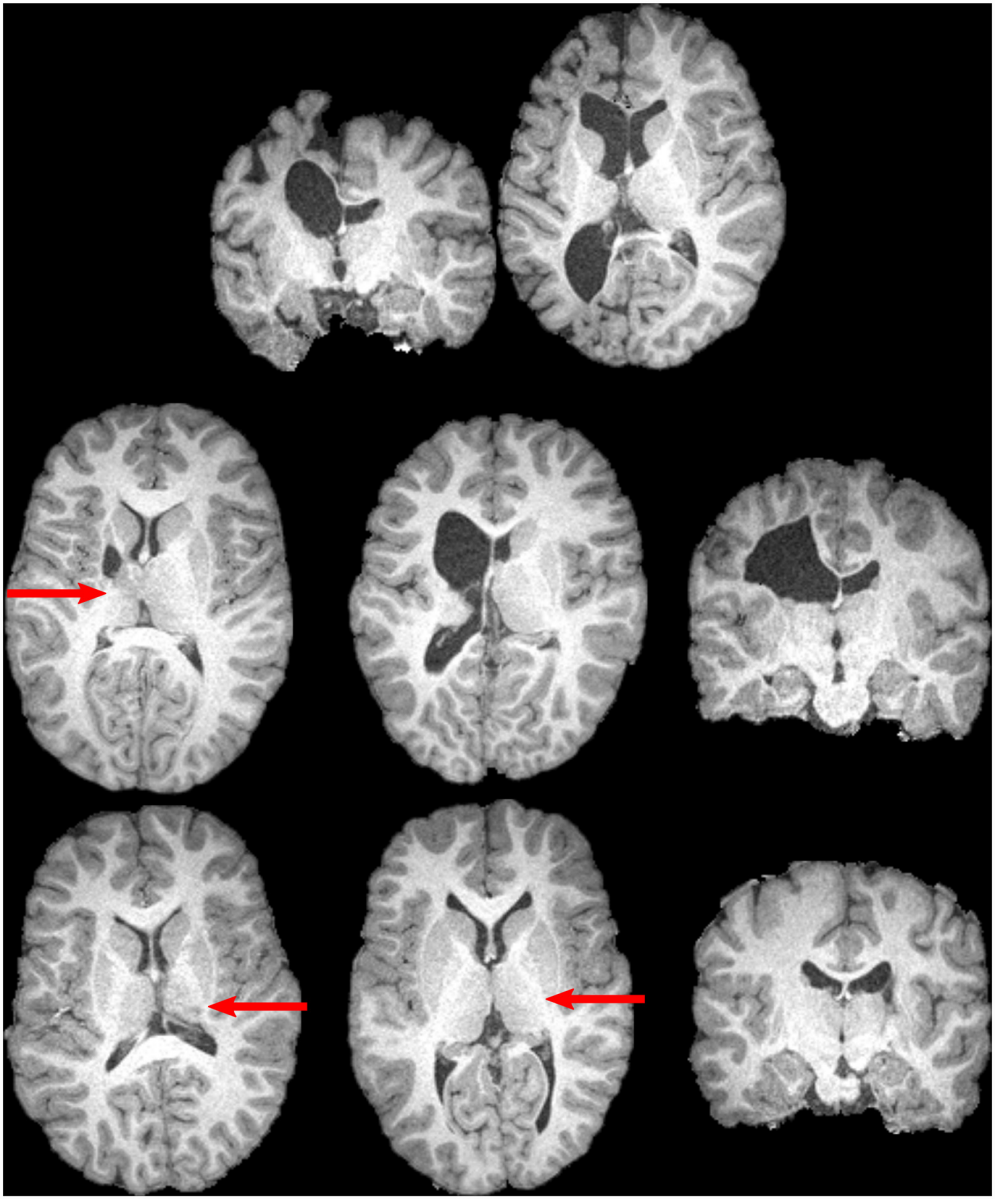
Examples of participants with unilateral cerebral palsy, for whom neurological abnormalities include distortion of the midbrain, and/or loss of contrast between the thalamus and posterior limb of the internal capsule. Subtler losses in contrast are indicated by arrows. Such abnormalities can make reliable delineation of these structures difficult to perform. The top row displays two slices from a single participant. The remaining images are from six different participants. All displayed participants contributed data that was utilized for one or more analyses.

This paper describes a surface-based fMRI protocol that performs reliably in people with moderate or significant pathology, and minimizes the aforementioned difficulties related to smoothing by eliminating reslicing. The resulting surface is used to both seed and anatomically constrain diffusion tractography. Rather than requiring high quality 3D ROIs of the midbrain, which can be difficult to obtain in the presence of pathology (Fig 2), the method optionally provides track categorisation through simple clustering algorithms that accept roughly-drawn 2D ROIs as hints. This method was demonstrated in a cohort of children with mild-moderate unilateral cerebral palsy, and the resultant diffusion measures were correlated with participants’ clinical characteristics. These results were compared with results obtained using a more standard voxel-based-fMRI + dMRI pipeline on the same dataset. The surface-based method was also run without track clustering to determine the contribution of this element to the quality of tractography.

## Methods

### Overview

Two pipelines were used to obtain corticomotor and thalamocortical tracks, from which measurements of fractional anisotropy (FA) and mean diffusivity (MD) were obtained. The *Surface* method utilized a simple tissue segmentation approach to generating a mesh. This mesh was then used in a surface-fMRI analysis that identified the sensorimotor area for each participant. Diffusion tractography was then seeded from this sensorimotor surface, and constrained by this mesh. Corticomotor and thalamocortical tracts were then delineated using a clustering algorithm. The *Voxel-Based* analysis used a standard SPM fMRI analysis in voxel space to identify the sensorimotor area. This area was then used to seed tractography, which was constrained by a brain mask. Corticomotor and thalamocortical tracks were then delineated using hand-drawn midbrain ROIs.

### Participants

**Fig 3:**
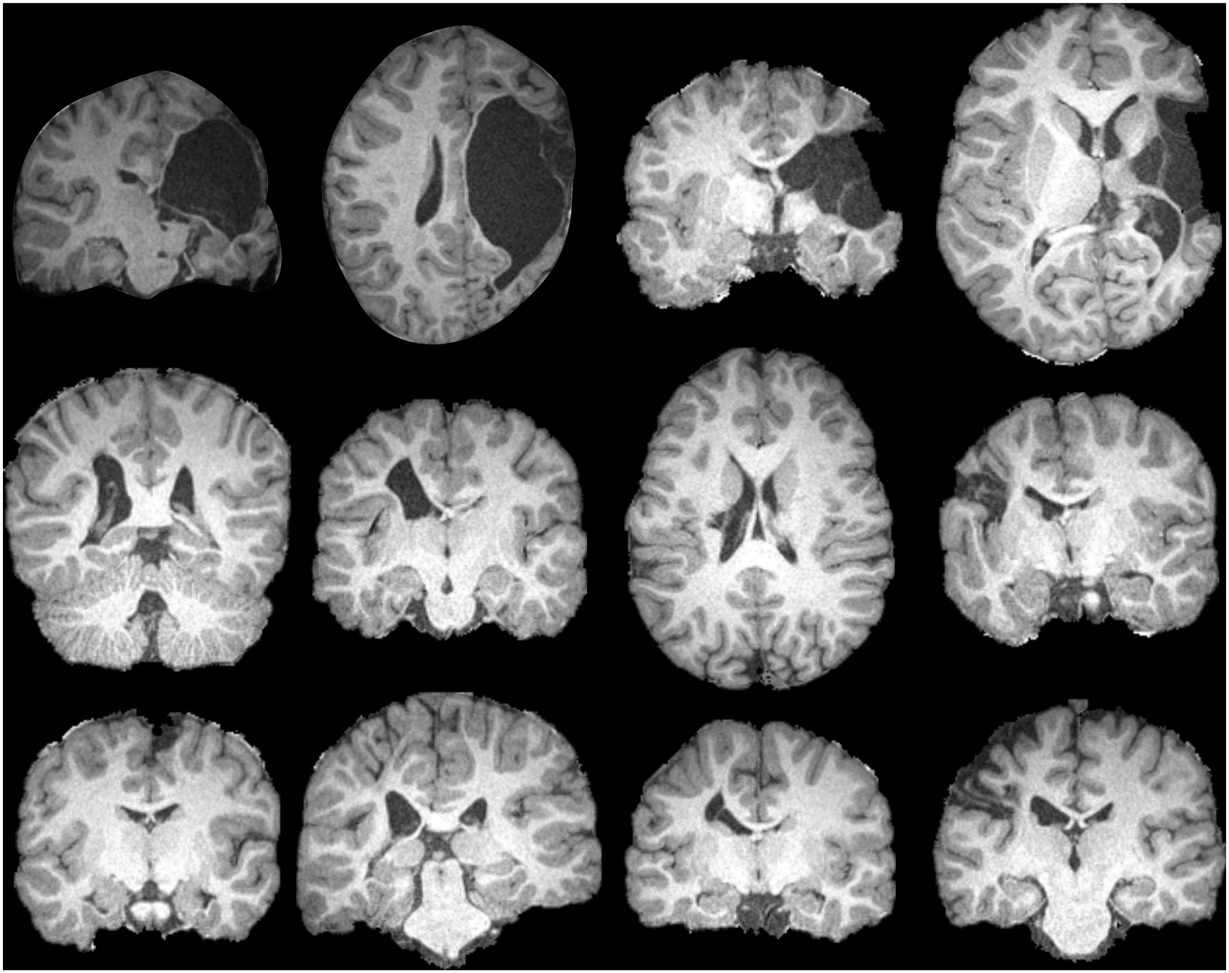
Examples of pathology seen in the enrolled cohort. The top row displays one coronal and one axial image from two participants with severe brain pathology. The remaining two rows display images of eight unique participants. No participants are duplicated from Fig 2. All displayed participants contributed data that was utilized for one or more analyses. These images, combined with those in Fig 2, are representative of the range of pathology severity seen across enrolled participants.

Imaging and clinical data were acquired as part of the Mitii clinical trial [15]. This analysis included data from 37 children (Mean age 11.7y; SD 2.7y), who had acceptable dMRI scans and did not exhibit behavioural issues that impaired data quality (e.g. repeated purposeful head movement) during fMRI or structural scans. All had a diagnosis of mild to moderate spastic-type unilateral cerebral palsy, with injury to the periventricular white matter (65%) or cortical deep grey matter (35%), a Gross Motor Function Classification System Level I (43%) or II, and Manual Abilities Classification scale (MACS) I (32%) or II. Diagnoses of CP subtype were made by a paediatric neurologist based on T1, T2 HASTE, and T2 TIRM volumes; the neurologist was blinded to clinical information. Detailed clinical characteristics are provided in Table 1. Representative examples of pathology are shown in Figs 2 and 3. Written informed consent was obtained from the parent or legal guardian of each child. Ethical approval was obtained by the Medical Ethics Committee of The University of Queensland (2011000608), The Royal Children’s Hospital Brisbane (HREC/11/QRCH/35), and the Cerebral Palsy Alliance Ethics Committee (2013-04-01). The Mitii study was registered with the Australian clinical trials register (www.anzctr.org.au): ACTRN12611001174976.

**Table 1:** Participant details for each stage of analysis.

Analysis Stage	N	Age	Left-sided Hemiplegia	Male	GMFCS I	MACS I	PWM	AMPS-M	JHFT-AI	COPM-P	AHA	MUUL
Enrolment	37	11.7±2.7	41%	51%	43%	32%	65%	1.29±0.37	0.46±0.28	4.50±1.27	70.1±14.8	85.9±16.9
Attempted	31	11.8±2.9	45%	45%	48%	32%	71%	1.32±0.36	0.44±0.27	4.58±1.26	70.8±13.2	88.5±12.3
Surface Succeeded	27	12.0±2.7	41%	44%	48%	37%	74%	1.37±0.33	0.39±0.25	4.65±1.18	73.8±10.7	90.8±9.9
Surface Correlations	24	11.6±2.4	44%	48%	50%	38%	79%	1.39±0.33	0.40±0.25	4.68±1.25	74±11.4	91.1±9.9
Voxelwise Succeeded	20	12.0±2.7	45%	50%	40%	30%	75%	1.26±0.31	0.45±0.28	4.54±1.19	71±12.6	89.9±10.8
Voxelwise Correlations	18	12.0±2.8	50%	50%	39%	28%	83%	1.26±0.30	0.48±0.28	4.53±1.26	70.7±13.3	89.5±11.0
Renalysis	16	12.0±3.0	44%	56%	38%	31%	88%	1.28±0.30	0.43±0.27	4.41±1.28	72.8±12.4	90.1±11.0

Left-sided hemiplegia refers to percentage of participants whose left arm was more impaired than their right. GMFCS I and MACS I refers to the percentage of patients scored as classification I for these scores; all others are level II. PWM refers to the percentage of participants with periventricular white-matter damage; all others had cortical deep grey matter injury. ‘Attempted’ rows include participant-datasets for whom fMRI analysis was attempted. ‘Succeeded’ include all datasets that were successfully processed through to diffusion metrics, inclusive. ‘Correlations’ rows include all participants included in correlation analyses (i.e. excludes participants with distinctively non-unilateral lesions). ‘Reanalysis’ row refers to the subset of data used during reanalysis of overlapping voxelwise and surface-based data. Ages and clinical scores are provided as mean ± standard deviation. All percentages are to 2 sf. Acronyms: GMFCS, Gross Motor Function Classification System; MACS, Manual Ability Classification System; PWM, periventricular white-matter damage; AMPS-M, motor component of the Assessment of Motor and Process Skills; MUUL, the Melbourne Unilateral Upper Limb Assessment; AHA, the Assisting Hand Assessment logit; JHFT-AI, the Jebsen-Taylor Hand Function Test asymmetry index; COPM-P, The Canadian Occupational Performance Measure performance score.

### Surface Method

**Fig 4:**
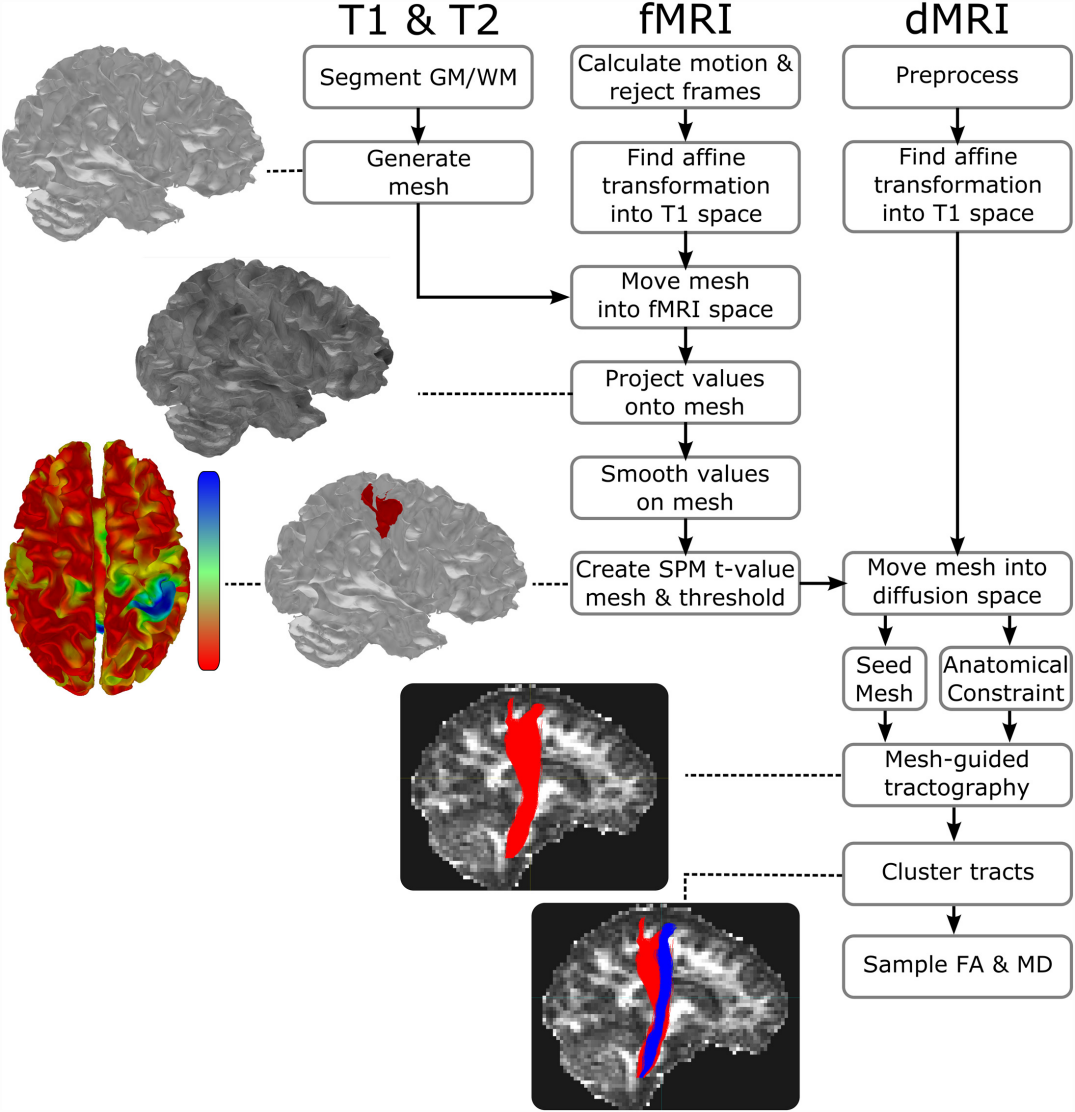
Summary of the pipeline for the Surface-based fMRI-guided dMRI tractography. Images and meshes associated with each major step depict real results from a single participant enrolled in this study. Major steps of the pipeline include tissue segmentation, mesh generation, projection of fMRI echo-planar image values onto the mesh, statistical fMRI calculation on the mesh, fMRI-seeded tractography, and tract clustering. See text for additional details.

The surface method pipeline is summarised in Fig 4.

#### Mesh Generation

A mesh of the grey matter-white matter interface was generated using a tissue segmentation driven by an expectation-maximization algorithm that has been described previously [16,17]. In this study, high resolution T1 MPR (TR/TE: 1900/2.32ms; 0.9mm isotropic) and T2 HASTE images (TR/TE 1500/81ms; 0.69 x 0.69 x 4mm) were used for tissue segmentation, and six age-appropriate atlases (from typically developing children) were utilized for each participant. Two Gaussians were fit to describe to gray-matter tissue intensities, and one Gaussian was fit to each of the other tissue classes. To improve this segmentation, a white-matter exclusion mask was generated by dilating the inverted brain mask three times. Clusters of voxels classed as WM were reclassed as gray matter if they both (i) overlapped this mask at any position and (ii) were smaller than 40 voxels in volume. The marching squares algorithm then produced a mesh of the gray-matter/white-matter interface with a mean vertex-to-neighbour-vertex distance of ~0.9mm. Meshes were in participant T1 space and checked visually before further analysis.

#### Surface-based fMRI

Two block-design task-based fMRI scans, each consisting of 90 echo planar images (TR/TE 3000/30ms; 3mm isotropic; full brain coverage), were collected for each participant in a single scan session on a Siemens Tim Trio 3T (Siemens, Erlangen, Germany) scanner. Distortion correction was applied online by the scanner. Each scan consisted of nine 30-second blocks, alternating between ‘stop’ and ‘move’ conditions. A visual cue consisting of the words ‘stop’ or ‘move’ was visible at all times, in addition to an auditory click that was delivered at 1Hz. Participants were instructed to tap their hand, by full extension at the wrist, in time with this cue in the ‘move’ condition, and to remain still during the ‘stop’ condition. Participants who were unable to perform a full-wrist extension due to physical impairment were asked to perform the largest tap that they could comfortably maintain (e.g. with fingers) without moving their forearm. Participants tapped using their more able hand in the first scan and more impaired hand during the second scan. We shall refer to these as the ‘able’ and ‘impaired’ hand tasks for brevity, but it should be noted that many participants displayed some degree of impairment in their more-able upper limb. These tasks were practiced out of the scanner immediately before the session began [15]. During the scan, an observer noted the quality of movements, the number of successful taps per block, as well as timing and degree of any body movements.

The long block lengths used in this scan eliminated the need for slice-timing corrections and reduced the potential impact of abnormal haemodynamic responses, which have been documented multiple times in participants with brain injuries [18–20].

**Motion and Projection:** The required coregistration between EPI images was estimated using *mcflirt* [21], and applied to each frame’s header (i.e. without reslicing). The registration between the T1 and mean EPI image used by *mcflirt* were calculated using the *epi_reg* script from FSL 5.0, which utilises boundary-based registration to generate rigid registration parameters [22]. The mesh was then moved into EPI space using these parameters, and visually inspected. To ensure analysis of gray-matter activation, the registered mesh was expanded outwards by 1mm before values were projected onto each vertex mesh using nearest-neighbour interpolation. The result for each dataset was a motion corrected cine-mesh of grey-matter EPI intensities.

Head motion larger than 2mm is common in children with cerebral palsy during fMRI scans, which can preclude standard fMRI analyses. Framewise displacement [23] was, therefore, calculated and frames with a framewise displacement above 0.9mm were rejected. Datasets with 20 or more rejected frames across the two fMRI scans were excluded from further analysis.

On the surface, our original node-to-node distance was around three times the EPI image resolution. Decimation was employed to halve the number of triangles in the mesh. This reduced computational complexity, whilst maintaining a sufficiently high-resolution surface to represent the underlying image data accurately. Surface-based smoothing of vertex values was then carried out using a Laplacian-Beltrami filter [24]. An 8mm FWHM smoothing kernel was selected based on the large cortical representation for hand and forearm movements described in anatomical studies of healthy participants [25] and fMRI studies of children with cerebral palsy [26,27].

**Analysis:** Required SPM 8 code was ported into the .NET framework and modified to accept meshes, rather than images, as input for all fMRI processing steps. Meshes were treated as two dimensional surfaces with anisotropic spacing between vertices. Due to frame rejection in the previous step, aspects of this code were changed to avoid implicit assumptions that frames were evenly spaced temporally. In line with a previous report [23], preliminary analyses demonstrated that the addition of translation (head-motion) parameters as GLM regressors in the presence of motion censoring made little difference to the degree of motion artefacts in participants with movement (visually assessed). Furthermore, in some instances these parameters sufficiently reduced the statistical power to the point where plausible significant activation shrank visibly in participants demonstrating weaker activation. Because of this, we included only rotation parameters as covariates-of-no-interest for our analysis. A high-pass filter of 120s and the standard SPM8 hemodynamic response function were used. Temporal autocorrelation was modelled using the standard white noise and autoregressive AR(1) model. Contrasts between ‘move’ and ‘stop’ conditions were calculated for the able- and impaired-hand scans separately, and the resulting standard parametric maps for each scan were thresholded at p<0.05 (FWE corrected) and binarised. Group analyses were not performed for the surface or voxel-based fMRI, as the purpose of the fMRI analysis was to locate the functionally relevant primary sensorimotor area for each individual, rather than interpret activation metrics.

**Filtering**: Significant activation was often seen in regions such as the supplementary motor area and cerebellum that were not of interest for this particular analysis. The largest plausible activation clusters near the presumed hand knob on the precentral gyri were retained and other activation clusters (including minor motion artefacts) were manually discarded (S1 Fig). Participants noted to have moderate degrees of motion artefacts in this step were rejected. The two resulting binarised SPM meshes for each participant were then combined with logical ‘OR’ for a final seeding mesh.

#### Tractography

We acquired a 64-direction high-angular resolution diffusion image sequence with b = 3000s/mm^2^ [28], whole brain coverage, and 2.34mm x 2.34mm x 2.5mm spatial resolution (NEX = 1). An extensive preprocessing procedure that has been described previously [29,30] was utilised to correct for image artefacts caused by cardiac pulsation, head motion, and image distortions. FA and MD maps were calculated using a copy of MRTrix 2.9 that was modified to utilise surfaces as masks during tractography. Constrained spherical deconvolution was used to estimate the fibre orientation distributions utilised for tractography.

The registration between the T1 and FA map was calculated using the *epi_reg* script from FSL. The seeding mesh was returned to the grey-matter/WM interface in T1 space, then registered to diffusion space using these parameters. At this stage, the seeding mesh was duplicated to provide an anatomically-constraining mesh (mesh ‘brain mask’). The seeding mesh was then shrunk inward 1mm at all points to ensure tracks started in the WM.

**Fig 5:**
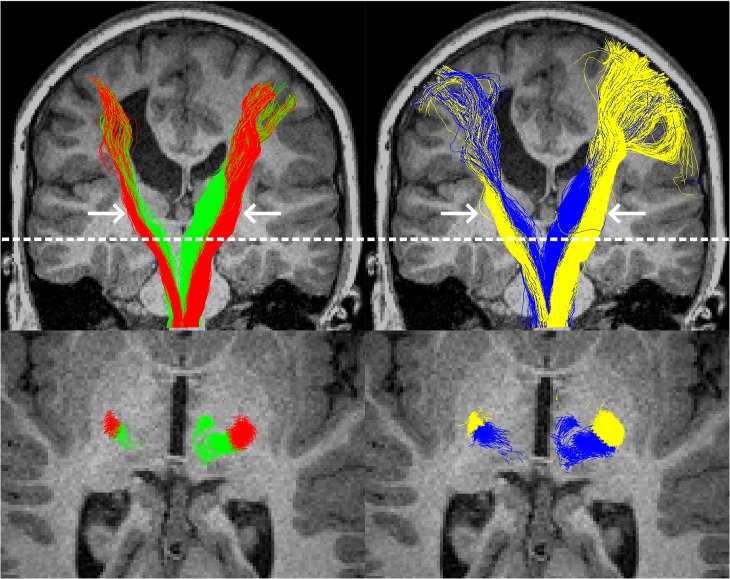
Example tractography from the surface-based (left column) and voxel-based (right column) methods, in a single participant with left-hemispheric pathology. The top row shows all tracks from all slices, with thalamocortical tracts in green or blue and corticomotor tracts in red or yellow. In this participant, the superior sections of the tracts were considerably more coherent when delineated with the surface-based method. The bottom row shows an axial slice at the level of the dotted white line. For this participant, at this level, track clustering (left) provided visually similar results to the region-of-interest based approach to track classification. Arrows indicate the approximate location of the midbrain regions of interest that were used to filter tracks.

As we were interested in both the thalamocortical and corticomotor tracks, two-dimensional inclusion ROIs of (1) the thalamus and (2) the posterior limb of the internal capsule were drawn with ITK SNAP [31] on a 1mm-isotropic track density image [32] at the most superior axial slice inferior to the corpus callosum (Fig 5). An inclusion ROI of the brainstem was drawn on the first axial slice inferior to the pons. ROIs were drawn by a researcher blinded to participants’ clinical scores. For each participant, a target of 20,000 tracks were generated that passed from the seeding region, through the either of the midbrain ROIs, then terminated in the brainstem ROI. A similarly-drawn two-dimensional ROI of the corpus callosum was used as an exclusion mask to exclude inter-hemispheric connections.

Seeding for probabilistic tractography took place from all triangles consisting of three neighbouring vertices that survived the statistical thresholding described earlier. Seeding was probabilistic and approximately uniform across the seeding area: the likelihood of each triangle being chosen to be seeded from on any given iteration was linearly-proportional to its surface area. The seed positions within each triangle were random and resultantly uniform. The initial direction for each track was inward, and perpendicular to the triangle from which it was seeded from. Tracks were terminated upon entering any voxel with an FA ≤ 0.1 or crossing any triangle in the anatomically-constraining mesh. A step size of 0.2mm and minimum curvature radius of 1mm were used.

**Semi-Automated Track Identification:** In preliminary analyses, manually drawn 2D posterior-limb-of-the-internal capsule and thalamic ROIs often proved ineffective for filtering thalamocortical from corticomotor tracks, primarily due to the partial overlap of such tracks in the midbrain. Manually drawing 3D ROIs, however, is extremely time consuming, especially in the presence of pathology or with large numbers of participants, and may be subject to human biases. To circumvent this problem, tracks were classed as either thalamocortical or corticomotor using the following two stage k-means clustering algorithm.

Track identification used a standard k-means clustering algorithm. Input was a single MRTrix track file containing all tracks passing from the assumed sensorimotor cortices, through the posterior limb of the internal capsule or thalamus, and to the brainstem inferior to the pons. Care was taken to draw the 2D midbrain ROIs as accurately as possible in this study due to the voxelwise analysis’ reliance on these ROIs. Experimentation suggested that clustering algorithm was very robust to roughly drawn ROIs, however, including when ROIs overlapped by several voxels in areas of uncertainty.

The first stage of the process split input into tracks into those in the left and the right hemispheres. The mean features of each cluster were initially set to the features from a random track. The algorithm was allowed a maximum of two clusters. Each track was converted into four features: the X and Y coordinates of a track’s first node (i.e. at the cortex), and the X and Y coordinates of the first node inside either of the midbrain ROIs, in standard space. Upon each iteration, the clustering algorithm assigned each track to the cluster that minimised the following cost function:

$$Cost\;=\sum_{i,f}{\left(t_{i,f}-\mu_{f}\right)}^{2}$$

Where *t*_*i*_ indicates track *i*, feature *f*, and *μ*_*f*_ indicates the mean value for feature *f* of the cluster in question. Convergence was defined as when no tracks were swapped between clusters on an iteration, or when 1000 iterations was reached. Clustering was attempted three times; results from the attempt with the lowest global cost were kept. The resulting clusters were automatically defined as either left- or right-hemispheric, based on their mean midbrain coordinates.

The second stage of the process separated motor from thalamic tracks, and was run on each hemisphere’s tracks separately. Twelve features were selected: the X and Y coordinates of the first node inside either of the midbrain ROIs; the X, Y, and Z coordinates of the 50^th^ node after (inferior to) the midbrain ROI; the X, Y, and Z coordinates of the node 50 nodes before the end of the track; the X and Y coordinates of the final node (i.e. brainstem); whether the track passed through the posterior limb of the internal capsule ROI (0 or 1); and whether the track passed through the thalamic ROI (0 or 1). The cost function used for this stage was:

$$Cost\;=\sum_{i,f}{\left(t_{i,f}-\mu_{f}\right)}^{2}\times w_{f}$$

Where *w*_*f*_ indicates weight for feature *f* for the current cluster, and other parameters have identical meaning to those in the previous equation. Weights were based predominantly on expected anatomy, but fine-tuned during preliminary analyses on four-participant datasets. For the thalamocortical cluster, all weights had a value of 1. For the corticomotor cluster, the weight for the midbrain Y coordinate was 0.49, reflecting the elongated shape of this tract at the midbrain; and weights were 1.69 for the X, Y, and Z coordinates at the 50^th^ node inferior to the midbrain, reflecting high spatial coherence at this position. Other weights for this cluster were 1. The thalamocortical cluster was initialised with the track closest to a point 5cm posterior to the centre of the brain. The corticomotor cluster was initialised with the track that was furthest from this posterior-central point at the midbrain ROI, with only those that passed through the medullary pyramids as eligible. The medullary pyramids in this context were defined as the anterior third of the brainstem ROI, or as the anterior third of all brainstem nodes if no tracks were found with the ROI criteria. Iteration and convergence criteria were identical to the previous stage. Clustering was attempted 20 times; results from the attempt with the lowest global cost were kept.

With exception of the manually drawn 2D ROIs, the entire track identification process was entirely automated and ran in under 30 seconds per 20,000 track dataset on a standard desktop PC.

### Voxel-Based Method

The voxel-based approach was carried out on the same dataset as the surface approach.

#### Voxel-Based fMRI

**Analysis.** The voxel based fMRI was carried out with a standard pipeline using SPM 8. As motion scrubbing is not available in this package, frames that were excluded from the Surface-based analysis due to movement were replaced with interpolated frames for this analysis. As with the surface analysis, an 8mm smoothing kernel was used. Other parameters and contrast were identical to the surface fMRI analysis. The resulting t-value maps were resliced into dMRI space using the same transform used for the surface analysis, before being thresholded at p < 0.05 (FWE corrected) and binarised.

**Filtering.** Filtering of significant clusters followed the same protocol as for the surface-based approach. For the voxel-based approach, activation maps were overlaid with structural (T1) images during this procedure to ensure correct filtering.

### Tractography

Tractography was carried out on the same diffusion images used for the surface-based analysis. The same inclusion ROIs were used as the surface based method to identify a target of 20,000 tracks passing from the cortex to the brainstem via the midbrain. Filtered fMRI activation from the previous step was used to seed tractography bi-directionally, with the brainmask used as a stop mask. Other parameters were identical to the surface-based method.

Tracks passing through the posterior limb of the internal capsule at any point were classed as corticomotor tracks; tracks passing through the thalamus and not the posterior limb of the internal capsule were classed as thalamocortical tracks.

### Tract Dispersion

One argument made for our surface-based approach was to reduce the number of non-relevant tracts included in tractography. If only tracks from a single white-matter bundle are identified, these tracks should be directionally coherent. To quantify this, we calculated tract dispersion for each motor and sensory tract as the mean square error in stepping direction at 20 node-positions that are evenly spaced across each track (i.e nodes at 0%, 5.3% … 94.7%, 100% of track length). Error here was defined as the geodesic difference from the mean direction (at that point of the track) across all tracks in the collection. Conceptually, if tracks are pointed in similar directions at equivalent nodes throughout their length, dispersion will be low. If tracks are commonly pointed in different directions at these equivalent positions, dispersion will be high. Mathematically:

$$\text{dispersion}\;=\;\frac{\sum_{n\;=\;1}^{20}\;\sum_{\text{i}}^{\text{t}}{\left(\text{Geo}\left(\theta_{n,i}-{\bar{\theta }}_{n},\phi_{n,i}-{\bar{\phi }}_{n}\;\right)\right)}^{2}}{20\text{t}}$$

where *n* is the node-position; *t* is the number of tracks in the collection; θ_*n*,*i*_ indicates inclination of direction at node-position n, track i; ${\bar{\theta }}_{n}$ indicates the mean inclination across all tracks at node-position *n*; ϕ_*n*,*i*_ indicates azimuth of direction at node-position *n*, track *i*; ${\bar{\phi }}_{n}$ indicates the mean inclination across all tracks at node-position *n*; and Geo is the function for calculating geodesic distance. Each spherical coordinate was calculated from the Euclidean coordinates, in standard space, of the node at node-position n, and the immediately following track node.

### Diffusion Metrics and Clinical Assessments

Diffusion metrics were calculated identically for the Surface and Voxel-Based approaches. FA and MD values were calculated with MRTrix 2.9 using CSD images. Each tract was split into superior (superior to the midbrain ROI) and inferior segments (inferior to the midbrain ROI), to provide measures for these locations separately. Asymmetry indices of sampled FA and MD measures were calculated the formula AI = (A-I)/(A+I), where A and I refer to the mean sampled values for the hemispheres opposite to the ‘able’ and ‘impaired’ hands, respectively. Asymmetry indices were adjusted for age and correlated with five clinical assessments carried out on the same day as scanning. These assessments were the Assessment of Motor and Process Skills motor component (AMPS-M) [33], a observational assessment of the quality of performance of activities of daily living; the Melbourne Unilateral Upper Limb Assessment (MUUL) [34], an objective measure of upper-extremity function; The Assisting Hand Assessment logit (AHA), an index of the impaired hand as an assisting hand in bimanual tasks; the Jebsen-Taylor Hand Function Test asymmetry indices (JHFT-AI), a test of uni-manual hand speed and dexterity, converted here into an asymmetry index; and The Canadian Occupational Performance Measure performance score (COPM-P), the participants self-reported satisfaction with their ability to carry out everyday tasks.

## Results

### Participants, fMRI, and Movement

Of the 37 children included in this investigation, four demonstrated clear head movement in time with hand tapping—a consequence of participants exerting substantial effort in order to achieve muscular contraction and/or an inability to dissociate hand from total body movement. Two additional children who were not noted as exhibiting excessive motion during scanning still demonstrated framewise displacement greater than 0.9mm in more than 20 frames. After exclusion of these participants, 31 children remained. Across the remaining datasets, the mean and median numbers of rejected frames were 4.6 and 1, respectively. The rejected frame counts did not correlate significantly with any clinical score (all p>0.05 and R^2^<0.1). Scans revealed large bilateral pathology in three of the remaining participants (despite being clinically assessed as unilateral CP). These participants were still processed to determine the success of the investigated methods in the presence of such pathology, but were excluded from final correlation analyses as asymmetry indices would be a poor reflection of tissue integrity in these instances.

#### Surface Method

**Fig 6:**
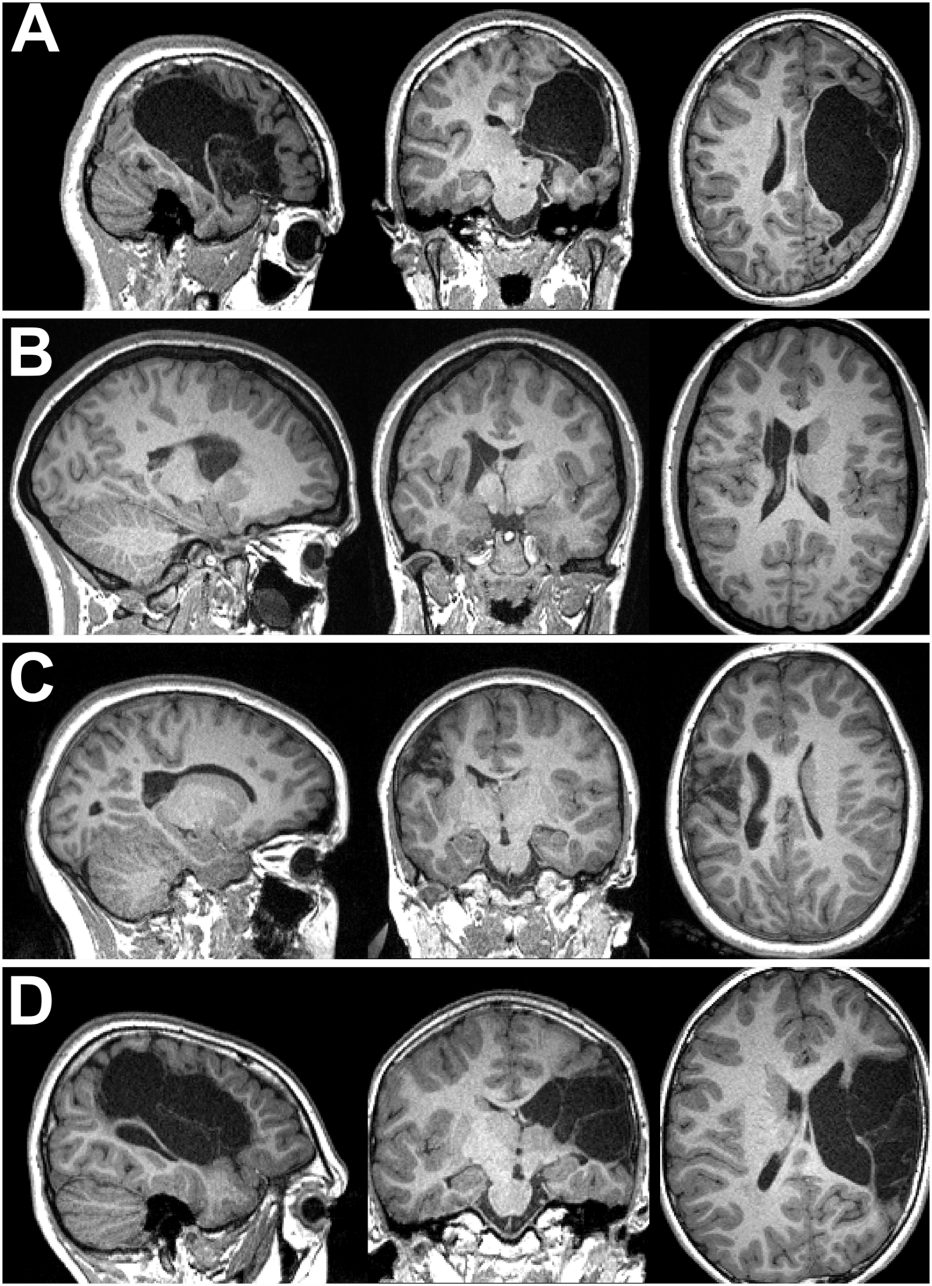
Examples of pathology and impacts on analyses. Row A: The child for whom a surface could not be generated. Tissue segmentation failed as the allowed magnitude of warping was insufficient to match any atlas, leading to cerebrospinal fluid being classed as grey and white matter. Voxelwise analyses were successful. Rows B and C: Two participants for whom surface analyses were successful, but voxelwise analyses failed to detect significant fMRI activation. Row D: A child with severe pathology for whom both voxelwise and surface analyses detected fMRI activation. Both methods found no corticomotor or thalamocortical tracks in the hemisphere with pathology; no genuine connections of this type were probably present.

Automated tissue segmentation succeeded in 30 of 31 participants, including several with moderate or severe pathology and both participants with distinct bilateral injury (Fig 6D). Of these, minor manual editing to delineate dura from brain tissue was required in two instances. Tissue segmentation failed in one case where severe pathology was present (Fig 6A); cost-function marking or manual delineation of pathology may have allowed segmentation to succeed in this case but was not attempted. Generation of the surface mesh from segmentation succeeded in all instances. Segmentation and mesh generation time took ~75mins per participant on a desktop PC.

**Fig 7:**
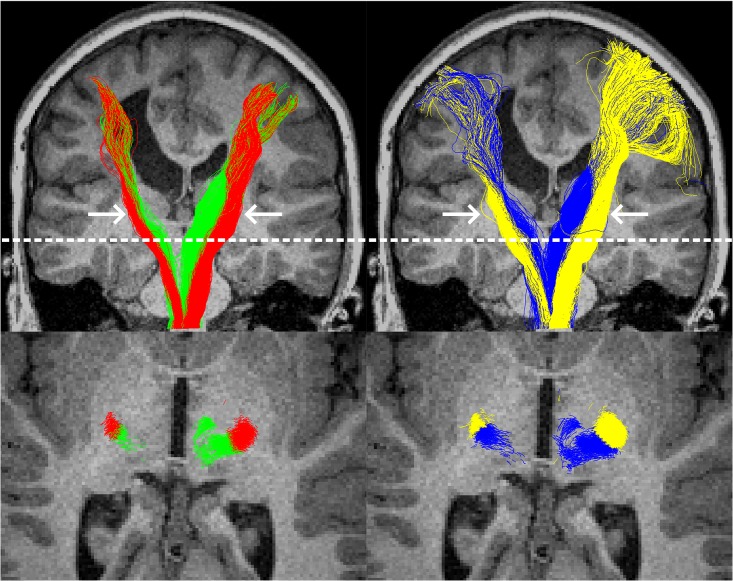
Typical significant fMRI activation detected through surface (top row) and voxelwise (middle and bottom rows) methods for tapping of the ‘impaired’ hand in a single participant. The middle and bottom rows show coronal, sagittal, and right-facing axial sections in the left, middle, and right columns respectively. Both methods show activation (red) in the approximate pre- and post-central gyri of the left hemisphere, and the right anterior lobe of the cerebellum. The voxelwise analysis resulted in approximately oval shaped activations that include grey-matter, white-matter, and cerebrospinal fluid. The surface-based method resulted in less-uniformly shaped activation patterns and two activation sites on the cerebellum.

Functional MRI analyses were completed successfully for the 30 remaining participants. Fig 7 shows an example of activation in the presumed sensorimotor cortex (S1M1) and anterior lobe of the cerebellum for a single participant during the ‘impaired’ hand task. Significant activation was seen in the region physically resembling the S1M1 for all of the 60 tasks across the 30 participants (Table 2). This was bilateral in seven instances (six during the able task). Significant activation was also seen in the supplementary motor area (16 participants), anterior lobe of the cerebellum (21 participants), and superior parietal lobule (7 participants). Such activation was discarded prior to tractography. Small areas of significant activation that were considered to be motion artefacts or statistical anomalies also appeared in thresholded results for eight participants, and were similarly discarded. In no instance were motion artefacts severe enough to justify rejection of the participant.

**Table 2:** Counts of Participants Displaying Significant Activation.

Surface Method (n = 30)						
	S1M1		SMA	Ant. Cerebellum	SPL	None
	Unilateral	Bilateral				
Able Hand Task	24	6	14	19	4	0
Impaired Hand Task	29	1	10	10	3	0
Either	30	7	16	21	7	0
Voxel Based Method (n = 30)						
	S1M1		SMA	Ant. Cerebellum	SPL	None
	Unilateral	Bilateral				
Able Hand Task	24	2	10	14	3	0
Impaired Hand Task	23	2	8	6	2	6
Either	29	3	14	16	4	6

Significant activation was defined as values of p<0.05 FWE in three or more connected vertices (Surface method), or one or more voxels after reslicing to diffusion space (Voxel-Based method), for five motor regions. ‘Either’ refers to the number of participants in which the specified activation pattern was seen for either task performed. None indicates that no significant activation was seen in any of these regions in either task. Abbreviations: Ant. Cerebellum, anterior lobe of the cerebellum; S1M1, sensorimotor cortex, comprising of the precentral and post central gyri; SMA, supplementary motor area; SPL, superior parietal lobule.

To alleviate concerns that tractography correlations could be the product of different degrees of fMRI activation, we calculated correlations between thresholded-fMRI activation area in the presumed S1M1 and clinical scores. Data from all 30 remaining participants were used. No informative correlations between clinical scores and impaired-hemisphere activation area, or activation-area asymmetry, were found (all R^2^≤0.15).

#### Voxel-Based Approach

Voxel-wise fMRI was completed acceptably in 30 of 31 participants: one participant-dataset was rejected due to the presence of severe motion artefacts. Significant activation was seen in the region physically resembling the S1M1 for 51 of the 60 tasks across the remaining participants (Table 2). This was bilateral in four instances (two during the able task). Compared with the surface method, clusters of significant activation were seen less frequently in the supplementary motor area, cerebellum, and superior parietal lobule (Table 2), but, visually, appeared to more frequently span multiple functional areas (e.g. both S1M1 and the supplementary motor cortex; data not shown). Thalamic activation was seen in two participants. Six participants displayed no significant activation during the impaired hand task (Fig 6). As our metrics were based on a ratio between hemispheres, nine participants who displayed significant S1M1 activation in only one hemisphere (across their two tasks) were excluded from further analysis.

No informative correlation was found between clinical scores and impaired-hemisphere S1M1 activation-volume, or activation-volume asymmetry (all R^2^≤0.10; n = 30). Results were similar when this analysis excluded the nine participants displaying unilateral-activation, with exception of activation volume asymmetry, which correlated weakly with JHFT-AI (R^2^ = 0.24).

### Tractography

For the surface method, no or very-few tracks were found on the side of the brain with pathology in three participants (Fig 6D). Based on inspection of structural MRIs, it was highly plausible that these participants were ipsilaterally-organised for one or both of these tracts. These participants were excluded from further analysis. Tractography and clustering in the remaining 27 participants was successful (visually assessed; Fig 5). It is noteworthy that clustering provided sensible results for one participant with pathology to the midbrain, and in one excluded participant who lacked thalamocortical tracks in the hemiplegic hemisphere (data not shown).

For the voxel-based approach, one participant was excluded from analysis as tractography was unable to find sufficient tracks on the side of the brain with pathology. This participant was likely to be ipsilaterally organised (Fig 6D). Tractography was successful in the remaining 20 participants.

Tract dispersion was calculated for each tract in the 18 participants for whom tractography was successful using both methods. Tracts generated by the voxel-based approach had significantly poorer (i.e. wider) dispersion than those generated by the surface-based method for the ipsilesional thalamocortical, contralesional thalamocortical, ipsilesional corticomotor, and contralesional corticomotor tracts (all p<0.01; Wilcoxon signed-rank test with Holm-Bonferroni Correction for multiple comparisons).

To calculate the influence of track clustering on tractography results, the surface method was re-run using midbrain ROIs to classify tracks. In this sub-analysis, no ipsilesional thalamocortical tracks were able to be identified for 5 of the 27 datasets. This ROI approach provided significantly poorer dispersion for the ipsilesional thalamocortical (p<0.01), contralesional thalamocortical (p<0.01), and contralesional corticomotor (p<0.05) tracts (Wilcoxon signed-rank test with Holm-Bonferroni Correction for multiple comparisons).

### Diffusion Metrics

#### Surface Method

After exclusion of the three participants with large bilateral injuries, 24 participant-datasets were available for correlations between age-adjusted diffusion metrics and clinical scores. For the upper corticomotor tract, both MD asymmetry and FA asymmetry correlated significantly (p<0.05) with all five clinical scores investigated (Table 3). For the upper thalamocortical tract, MD asymmetry correlated significantly (p<0.05) with all five clinical scores investigated, but FA asymmetry was not predictive of clinical scores. FA asymmetry of the lower corticomotor tract correlated significantly with AMPS-M (R^2^ = 0.44) and JHFT-AI (0.33), and approached significance correlating weakly with AHA logit (R^2^ = 0.24, p = 0.06), but not with other clinical scores. Clinical scores did not correlate with FA asymmetry of the upper or lower thalamocortical tract, nor MD asymmetry of either lower tract.

**Table 3:** Correlations (R^2^) between clinical scores and age-adjusted diffusion metrics recorded via tractography for two tracts superior to the midbrain ROIs.

	Superior Thalamocortical		Superior Corticomotor	
	FA	MD	FA	MD
	Surface Method (n = 24)			
AMPS-M	ns	0.33	0.43	0.26
JHFT-AI	ns	0.48	0.37	0.55
COPM-P	0.23†	0.33	0.28	0.26
AHA	ns	0.41	0.34	0.42
MUUL	ns	0.47	0.34	0.46
	Voxelwise Method (n = 18)			
AMPS-M	ns	Ns	ns	ns
JHFT-AI	ns	Ns	0.44	ns
COPM-P	ns	Ns	ns	ns
AHA	ns	Ns	0.27†	ns
MUUL	ns	Ns	ns	ns

Correlation analyses included all participant-datasets possible. ‘ns’ indicates that correlations were not statistically significant (p≥0.05).

^†^ indicates values that were not significant but for which p<0.1.

Abbreviations: AHA, The Assisting Hand Assessment logit; AMPS-M, Assessment of Motor and Process Skills motor component; COPM-P, The Canadian Occupational Performance Measure performance score; FA, fractional anisotropy; MD, mean diffusivity; MUUL, the Melbourne Unilateral Upper Limb Assessment; JHFT-AI the Jebsen-Taylor Hand Function Test asymmetry index.

#### Voxelwise Method

After exclusion of the participants with large bilateral injuries, 18 participant-datasets were available for correlation between age-adjusted diffusion metrics and clinical scores (Table 3). Significant correlations were only found between JHFT-AI and FA asymmetry, in the upper (R^2^ = 0.44; p = 0.01) and lower (0.35; 0.04) corticomotor tracts. All other correlations were not significant, although correlations between upper corticomotor FA asymmetry and AHA (R^2^ = 0.27; p = 0.07), and lower thalamocortical FA and JHFT-AI (0.28; 0.09) approached significance.

#### Analysis with Identical Participants

To determine whether differences between the surface and voxelwise methods were simply due to a difference in participants or participant numbers, correlation analyses were repeated with only the 16 participants with unilateral lesions who were able to be analysed by both methods. Fourteen of these participants had periventricular white matter injury. In this sub-analysis, for the surface method, all correlations between clinical scores diffusion metrics in the upper tracts strengthened considerably (Table 4), as did the correlation between lower corticomotor FA and JHFT-AI (R^2^ = 0.39), but the correlation between lower corticomotor FA and AMPS-M weakened (R^2^ = 0.31; p = 0.08). Other lower-tract correlations remained non-significant. For the voxelwise analysis, correlations remained for upper corticomotor FA asymmetry with JHFT-AI (R^2^ = 0.49; p = 0.01) and AHA remained (0.38; 0.05), and JHFT-AI and FA of the inferior corticomotor tract reached significance (0.46; 0.02), but all other correlations remained non-significant (Table 4). In this 16-participant group, the number of fMRI frames rejected due to motion was not significantly correlated with any clinical score.

**Table 4:** Correlations (R^2^) between clinical scores and age-adjusted diffusion metrics recorded via tractography for two tracts superior to the midbrain ROIs.

	Superior Thalamocortical		Superior Corticomotor	
	FA	MD	FA	MD
	Surface Method			
AMPS-M	0.31†	0.50	0.73	0.50
JHFT-AI	ns	0.58	0.49	0.71
COPM-P	0.30†	0.52	0.37	0.54
AHA	ns	0.54	0.48	0.62
MUUL	ns	0.61	0.43	0.64
	Voxelwise Method			
AMPS-M	ns	ns	ns	ns
JHFT-AI	ns	ns	0.49	ns
COPM-P	ns	ns	ns	ns
AHA	ns	ns	0.38	ns
MUUL	ns	ns	ns	ns

Correlation analyses used the 16 participant-datasets available to both methods. ‘ns’ indicates that correlations were not statistically significant (p≥0.05).

^†^ indicates values that were not significant but for which p<0.1.

Abbreviations: AHA, The Assisting Hand Assessment logit; AMPS-M, Assessment of Motor and Process Skills motor component; COPM-P, The Canadian Occupational Performance Measure performance score; FA, fractional anisotropy; MD, mean diffusivity; MUUL, the Melbourne Unilateral Upper Limb Assessment; JHFT-AI the Jebsen-Taylor Hand Function Test asymmetry index.

## Discussion

In this study, a novel data-processing pipeline was demonstrated that allows diffusion metrics to be obtained from participants with moderate to severe brain pathology in functionally-relevant regions of the thalamocortical and corticomotor tracts. This pipeline utilised a surface-based fMRI analysis to replace often-problematic parcellation, and minimized cross-sulcal smoothing present in standard fMRI analyses. This mesh was then used to both seed and anatomically constrain tractography that was subsequently dissected into tracts using a clustering algorithm. When applied to children with unilateral cerebral palsy, plausible correlations between diffusion metrics and functional ability were demonstrated, particularly for the superior aspect of the tracts.

### Method Comparison

The surface method was able to successfully process substantially more datasets (27) than the voxelwise method (20). This was primarily due to an apparently heightened sensitivity to fMRI activation (Table 2), reflected here by more-frequent detection of activation in supplementary areas, and in S1M1. Similar findings of heightened sensitivity have been reported in other comparisons of surface- and voxel-based analyses [35,36].

The surface method was also able to consistently find correlations between symptom severity and diffusion metrics in the superior aspect of the both corticomotor and thalamocortical tracts, but equivalent correlations using the voxelwise method were mostly absent. This was not due to participant numbers or different groups of participants as no obvious bias in dataset rejection in terms of age or ability was identified (Table 1), and reanalysis of these data using matched datasets strengthened correlations for the surface-based approach substantially more than those of the voxelwise approach (Table 4). One potential source of this difference may have been spatial sensitivity: areas of significant activation appeared qualitatively to be better restricted to within the S1M1 in the surface-based approach than rather than the voxel-based approach. This factor was not able to be quantified here, as this would require atlas-based parcellation, but better spatial specificity for surface or surface-like methods have been reported in several other studies [14,36–38]. Tracts elucidated using the surface method were also significantly more coherent, indicating that they each better represent a single population of fibres, than those generated via the voxelwise method. This was likely not only due to the tractography seeding, but also due to the clustering method, which resulted in superior track dispersion compared to ROI-based filtering. The surface-based tractography may have also played a role by preventing tracks from crossing sulcal boundaries in locations where the FA cut-off was undermined by partial volumes of grey and white matter (Fig 5).

It may be argued that the primary issue with the voxelwise analysis is the use of 2D, rather than 3D, midbrain ROIs. It is certainly possible that these results may have improved with 3D ROIs, but in any dataset where pathology prevents parcellation, these 3D ROIs must be manually delineated, which is very time consuming and difficult to perform accurately, even with super-resolution track density images. Even with 3D ROIs, the best method to classify tracks is not obvious, as the majority of thalamocortical tracks will also pass through voxels containing the posterior limb of the internal capsule at some point. Better voxelwise tractography may have resulted from a smaller fMRI blurring kernel, but the sensitivity of the fMRI analysis will likely have also suffered, resulting in rejection of even more participants than was seen here. Higher statistical thresholds are an alternative approach to constraining fMRI but, again, reduce the sensitivity of the fMRI analysis and may result in additional participant rejection. Tailoring statistical thresholds to each individual are a related approach but raises issues of data equivalency when data is pooled. For example, if one participant must have a threshold set at p<0.05 FWE, but another must have it as low as p<0.005 FWE, is the resultant tractography functionally equivalent? Surface based methods, however, provide equal-or-better statistical power than voxel-based smoothing [36], without such issues. For this reason we believe that surface smoothing is more appropriate for group-wise studies.

### Clinical Findings

In this study, significant correlations were found between five clinical scores of motor ability (AMPS-M, JHFT-AI, COPM-P, AHA and MUUL) and MD of the superior (i.e. cortical) section of both the thalamocortical and corticomotor tracts (Table 4). These scores also correlated with FA of the upper corticomotor tract, but not of the upper thalamocortical tract. Previous studies of unilateral cerebral palsy in non-overlapping subject cohorts have reported moderate correlations between FA of these tract sections and AHA [30], as well as between corticothalamic tract streamline-count asymmetry and JHFT, MUUL, and AHA [9]. Our findings here contrast with the latter of these studies, which concluded that damage to the sensory tracts may hold more importance to clinical outcomes than damage to the motor tracts. Rather than contradicting the hypothesis that thalamocortical tracts are ‘more influential’ for function than motor tracts, this discrepancy more likely highlights that this condition is highly heterogeneous, and complex. I.e. the relative influences of lesions depend on additional factors such as the lesion’s type, specific location, and timing. The results presented from the two methods used here also highlights how different quantification methods can have distinctly different patterns of sensitivity. It is worthy of note that our analysed data came overwhelmingly from participants with unilateral periventricular white-matter injury (Table 1), and so results may not be generalizable to other forms of CP.

There were very limited correlations found between functional clinical scores and diffusion metrics of the inferior section of the tracts investigated. This is an interesting result, considering the strong correlations found for the superior aspects of the tracts. In one respect, this is not unexpected as all participants in this study had either diffuse axonal injury, or injuries predominantly at or superior to the level of the midbrain. If taken at face value, this would indicate that upper-tract damage, long term, is not strongly reflected further along those tracts, as may be expected from Wallerian degeneration or constrained neurological development. An alternative explanation is that the tensor diffusion metrics used here are not sufficiently sensitive to measure such differences, especially when measuring voxels that include ‘unimpaired’ fibre populations. Some evidence for this hypothesis was identified using apparent fibre density [39] as a metric, but due to uncertainties regarding the effects of intensity normalisation, we did not formally include this metric in this analysis. More advanced measures, such as ‘SIFT’ed track counts [40] are one way that this could be resolved, but these currently rely on full-brain tractography and require midbrain parcellation, which is not currently possible to automate in participants with the levels of pathology seen here. A final possibility is that these structures are impaired more bilaterally than more superior aspects of the tracts, limiting the sensitivity of asymmetry measures. One earlier study of unilateral cerebral palsy has provided some evidence for this hypothesis, finding that periventricular WM lesions are often associated with bilateral cortical or deep grey-matter lesions [41].

### Strengths and Limitations

One potential criticism of fMRI-dMRI methods, including these, is that acceptable quality functional scans are required for each participant, which can be difficult to achieve, and so such methods offer little-to-no advantage over the traditional parcellation-based approach. In our experience, acquiring most types of MRI in children with simultaneous learning and physical impairments can be a challenge as such disabilities can impair their ability to remember and follow instructions. In this study, four of the six excluded participants were excluded due to unconsciously nodding while tapping, a consequence of participants exerting excessive effort to achieve meaningful isolated muscular contraction. The issue of movement was thus primarily an instructional one that may be preventable in future studies by allowing extra practice time before scan sessions, or more dedicated preparation procedures [42]. These rates of data rejection are still lower than our earlier attempts to parcellate brains using Freesurfer, where we were unable to successfully process ~30% of participants with only mild to moderate pathology. Another option is manual parcellation, but this is costly in terms of time, may introduce biases or errors. These issues aside, the primary advantages to fMRI-based seeding are that they enable investigation into participants with obvious pathology, who would otherwise be continually excluded from analyses. Functional MRI based seeding also theoretically provides higher specificity in tract identification than parcellation-based approaches. This is not only because fMRI tasks can be designed to provide specific subregions (e.g. the hand knob), but also because there is little certainty that regions defined by parcellation accurately identify *only* the desired functional areas when used in brains that have undergone atypical development or reorganisation. A final option is to use hand-drawn ROIs of the brainstem and midbrain, and track toward the cortex without use of any cortical ROIs. For identification of the entire corticomotor tract this is the obvious solution, though a degree of stray-track pruning may be required if tract metrics wish to be measured. There are numerous situations in which this is not sufficient however, such as studies looking at the relationship between upper limb abilities and diffusion metrics, or attempts to measure rehabilitation-evoked plasticity. In such studies, it is critical to identify the location specific to the upper-limb (or other body part) in question. Failure to do so can undermine sensitivity and biological interpretability of any resultant metrics.

A related criticism is that task fMRI results can be variable [13], and so could lead to differences in tractography that do not exist anatomically. We concede that single-participant activation maps based on a single time point are prone to such variability. Head motion can often correlate with participant ability [43], but was controlled for to the best of our ability in the present study. Task performance may also affect fMRI activation maps [13], but we aimed to minimise any such effects by utilising a very simple task and ensuring adequate task performance for each participant. Activation volume and area did not correlate meaningfully with clinical scores, so are unlikely to have biased diffusion results. Even with such controls, all fMRI-based seeding methods, particularly when applied to participant data that cannot be pooled, are at risk of fMRI variability leading to spurious correlations. This must be weighed up against the opportunity fMRI provides to perform tractography in datasets that otherwise would be discarded from atlas-based analyses. Regarding our method comparison, however, we consider it unlikely that the surface-based method outperforming the other in almost all aspects was simply a product of biased seeding. This is because these methods utilised identical raw data, and so we should expect task-difficulty differences or other sources of variation to equally influence both methods investigated. I.E it is unclear how sources of fMRI variability could artificially generate correlations between diffusion and clinical-test metrics for *only one* of these methods.

Finally, brain pathology can be accompanied by haemodynamic abnormalities. Quantifying these is potentially critical for a study with a difficult task, when differences in fMRI patterns intend on being directly interpreted. It is also important for event-related designs where changes between action/rest blocks are frequent. The present study, however, involved a very simple task with very long blocks. This design minimises the influence of abnormal haemodynamic response curves, and was only used to identify the ‘hand’ region of the S1M1. Again, as the activation area and volume did not correlate with any clinical test recorded, this design likely fulfilled its purpose, without introducing confounds that could lead to type-I errors.

## Conclusions

Investigating diffusion characteristics of people with moderate brain pathology is difficult or impossible to perform with standard parcellation-based approaches, and has led to fMRI-seeding of dMRI data. In this work, a novel highly-automated fMRI-dMRI fusion pipeline that identified distinct correlations between functional clinical scores and dMRI metrics of sensorimotor tracts in children with unilateral cerebral palsy was presented. These relationships were substantially stronger when delineated with this approach than when delineated with a standard voxelwise fMRI-seeding approach, and were minimally apparent in the inferior aspect of these tracts, where pathology was absent. The approach demonstrated here may prove valuable to studies of brain pathology, especially where sensitive analyses are required, such as the measurement of neuroplastic changes induced by intervention. The presented method may also ultimately prove useful for presurgical planning—for example, planning of neurosurgery aiming to treat intractable epilepsy in children with pre-existing brain injuries.

## Supporting Information

S1 Fig Illustration of functional MRI motion artefact and/or supplementary-activation rejection. Four participants (one per column) with motion artefacts are shown. The third column additionally displays activation of the supplementary motor area. Selected activation is shown in red, according to the criteria set out in Methods. Rejected activation is shown in blue. Note how only unambiguous activation within the major cluster of activation is selected in each instance. For surfaces in the top row, the front left of each image represents the anterior left of that participant. For surfaces in the second row within the first and second columns, a view from behind the participants are used: left of the image is left of the participant; top of the image is superior. The ‘sagittal’ view in the second row, third column, shows the left side of the brain; the left of the image is anterior. The bottom right image shows a view of the underside of the brain; the top of the image is anterior; the left of the image is the patient’s right.
(DOCX)
