# Correction: Surface-Based fMRI-Driven Diffusion Tractography in the Presence of Significant Brain Pathology: A Study Linking Structure and Function in Cerebral Palsy

**DOI:** 10.1371/journal.pone.0162271

**Published:** 2016-08-30

**Authors:** Lee B. Reid, Ross Cunnington, Roslyn N. Boyd, Stephen E. Rose

Fig 7 appears incorrectly in the published article. Please see the correct Fig 7 and its caption here.

**Fig 7:**
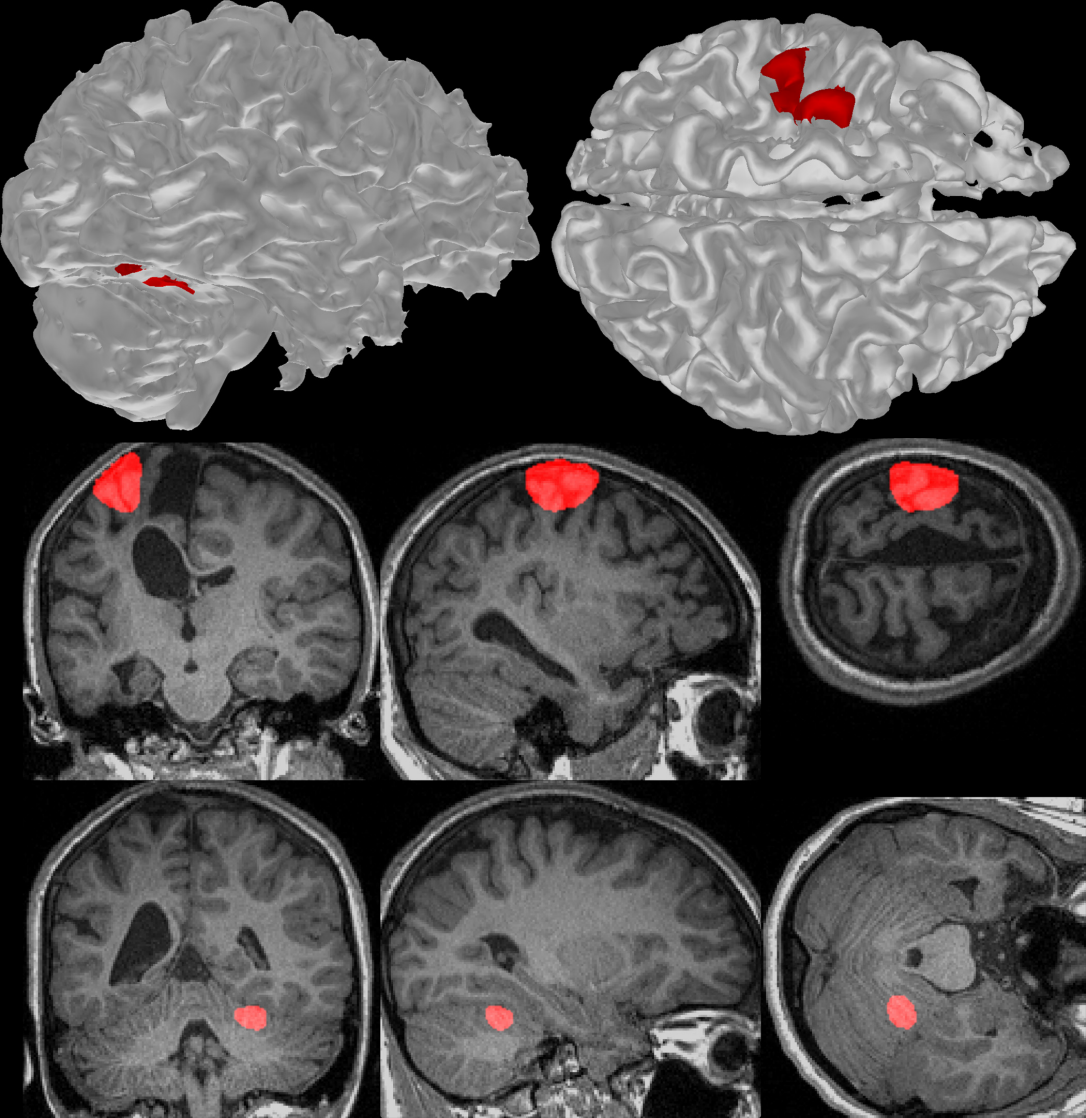
**Typical significant fMRI activation detected through surface (top row) and voxelwise (middle and bottom rows) methods for tapping of the ‘impaired’ hand in a single participant.** The middle and bottom rows show coronal, sagittal, and right-facing axial sections in the left, middle, and right columns respectively. Both methods show activation (red) in the approximate pre- and post-central gyri of the left hemisphere, and the right anterior lobe of the cerebellum. The voxelwise analysis resulted in approximately oval shaped activations that include grey-matter, white-matter, and cerebrospinal fluid. The surface-based method resulted in less-uniformly shaped activation patterns and two activation sites on the cerebellum.
