# Supplementary material for: Surface-Based fMRI-Driven Diffusion Tractography in the Presence of Significant Brain Pathology: A Study Linking Structure and Function in Cerebral Palsy
Source: PLoS One. 2016 Aug 3;11(8):e0159540. doi: 10.1371/journal.pone.0159540 (PMC4972431; doi:10.1371/journal.pone.0159540)
Supplement: S1 Fig — Four participants (one per column) with motion artefacts are shown. The third column additionally displays activation of the supplementary motor area. Selected activation is shown in red, according to the criteria set out in Methods. Rejected activation is shown in blue. Note how only unambiguous activation within the major cluster of activation is selected in each instance. For surfaces in the top row, the front left of each image represents the anterior left of that participant. For surfaces in the second row within the first and second columns, a view from behind the participants are used: left of the image is left of the participant; top of the image is superior. The ‘sagittal’ view in the second row, third column, shows the left side of the brain; the left of the image is anterior. The bottom right image shows a view of the underside of the brain; the top of the image is anterior; the left of the image is the patient’s right. (DOCX) [file pone.0159540.s001.docx]

# Supporting Materials

## S1 Activation Selection Examples

*
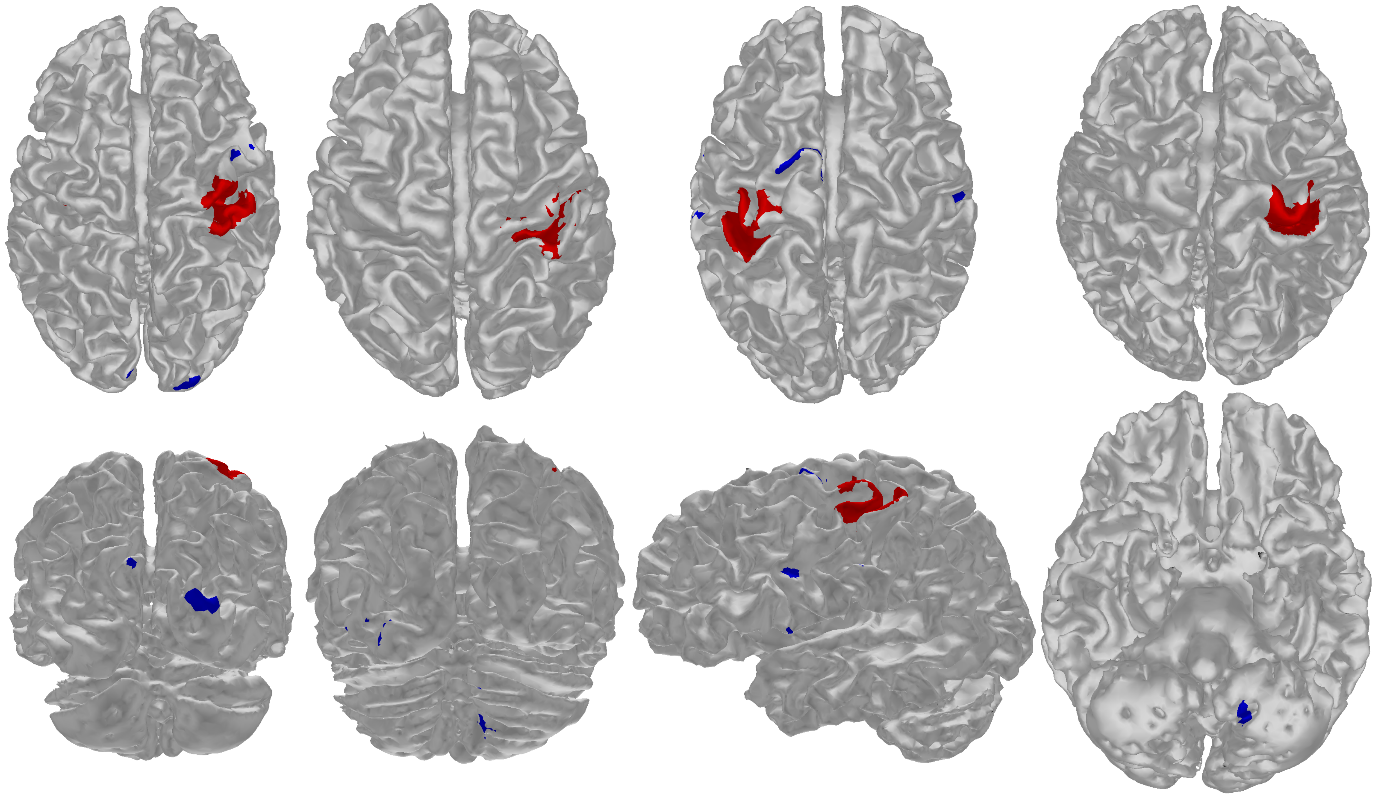
*

S1 Fig. Illustration of functional MRI motion artefact and/or supplementary-activation rejection. Four participants (one per column) with motion artefacts are shown. The third column additionally displays activation of the supplementary motor area. Selected activation is shown in red, according to the criteria set out in Methods. Rejected activation is shown in blue. Note how only unambiguous activation within the major cluster of activation is selected in each instance.
For surfaces in the top row, the front left of each image represents the anterior left of that participant. For surfaces in the second row within the first and second columns, a view from behind the participants are used: left of the image is left of the participant; top of the image is superior. The ‘sagittal’ view in the second row, third column, shows the left side of the brain; the left of the image is anterior. The bottom right image shows a view of the underside of the brain; the top of the image is anterior; the left of the image is the patient’s right.
